# Complexin-1 and Foxp1 Expression Changes Are Novel Brain Effects of Alpha-Synuclein Pathology

**DOI:** 10.1007/s12035-014-8844-0

**Published:** 2014-08-12

**Authors:** Suzana Gispert, Alexander Kurz, Nadine Brehm, Katrin Rau, Michael Walter, Olaf Riess, Georg Auburger

**Affiliations:** 1 Exp. Neurology, Goethe University Medical School, Building 89, Theodor Stern Kai 7, 60590 Frankfurt am Main, Germany; 2 Institute of Medical Genetics and Applied Genomics, University of Tuebingen, 72076 Tübingen, Germany

**Keywords:** Parkinson’s disease, Alpha-synuclein, Midbrain/brainstem expression changes, Presynaptic vesicles, Complexin-1, Foxp1

## Abstract

As the second most frequent neurodegenerative disorder of the aging population, Parkinson’s disease (PD) is characterized by progressive deficits in spontaneous movement, atrophy of dopaminergic midbrain neurons and aggregation of the protein alpha-synuclein (SNCA). To elucidate molecular events before irreversible cell death, we studied synucleinopathy-induced expression changes in mouse brain and identified 49 midbrain/brainstem-specific transcriptional dysregulations. In particular complexin-1 (*Cplx1*), *Rabl2a* and 14-3-3epsilon (*Ywhae*) downregulation, as well as upregulation of the midbrain-specific factor forkhead box P1 (*Foxp1*) and of *Rabgef1*, were interesting as early mRNA level effects of alpha-synuclein triggered pathology. The protein levels of complexin-1 were elevated in midbrain/brainstem tissue of mice with A53T-SNCA overexpression and of mice with *SNCA*-knockout. The response of CPLX1 and *Foxp1* levels to SNCA deficiency supports the notion that these factors are regulated by altered physiological function of alpha-synuclein. Thus, their analysis might be useful in PD stages before the advent of Lewy pathology. Because both alpha-synuclein and complexin-1 modulate vesicle release, our findings support presynaptic dysfunction as an early event in PD pathology.

## Introduction

Parkinson’s disease (PD) is the second most frequent age-associated brain degeneration disorder, affecting about 1 % of the population over 65 years of age. The PD-specific progressive movement deficit is mostly due to the severe affliction and cell death of midbrain nigrostriatal dopaminergic neurons [1]. Surviving neurons in vulnerable regions exhibit aggregates predominantly consisting of the protein alpha-synuclein, which are visualized as Lewy neurites and Lewy bodies, both in sporadic late-onset and most familial early onset PD variants [2].

Autosomal dominant PD with early clinical manifestation was observed in rare families, leading to the identification of alpha-synuclein (*SNCA*) protein missense mutations such as A53T (termed the PARK1 variant) and of *SNCA* gene duplication/triplication events (PARK4 variant) as the strongest causes of this pathology [3, 4]. Further recruitment of Parkinson’s families enabled identification of a list of disease genes responsible for monogenic PD [5]. In addition, recent characterization of very large collectives of late-manifesting sporadic PD cases through genome-wide allele association studies (GWAS) identified two regions on chromosome 4 (*SNCA* and *GAK/CPLX1* loci) that contain genetic variants predisposing to multifactorial PD [6]. Variations in the *SNCA* gene 3′-untranslated region (3′-UTR) and its promoter correlated strongly with PD risk [7].

Αlpha-synuclein is physiologically concentrated in axon terminals. It is associated with the lipid membranes of synaptic vesicles and interacts with synaptobrevin, a component of the SNARE complex, mediating vesicle exocytosis and neurotransmitter release [8]. Its toxic gain-of-function leads over time to impaired synaptic vesicle release and synaptic failure [9, 10]. Current investigations aim to elucidate alpha-synuclein-triggered pathology, concentrating on disease stages before the occurrence of irreversible cell loss, when neuroprotective therapies might still be efficacious.

Here, we focused on two independent mouse lines of inbred FVB/N background with ~1.5-fold overexpression of human A53T-alpha-synuclein in nigrostriatal dopaminergic neurons under control of the heterologous neuron-specific prion-promoter. A53T-alpha-synuclein overexpression in these mice occurs in presynaptic nigral dopaminergic neurons and presynaptic cortical glutamatergic neurons, but not in postsynaptic striatal neurons. These mice display apparently normal movements at age 6 months, but progress to significantly impaired spontaneous locomotion by age 18 months, despite the absence of neuronal loss in the nigrostriatal projection [11]. Previous expression profiling in these mice identified a *Homer-1a* transcript dysregulation throughout the brain and a 14-3-3 epsilon protein upregulation selectively in the striatum as molecular effects of alpha-synuclein triggered pathology. The alterations in these signalling molecules were temporally correlated with reduced striatal dopamine release and deficient long-term depression [12–14, 9]. To gain insight into the mechanisms underlying the impairment in vesicle exocytosis and neurotransmitter release, we surveyed progressive expression changes in midbrain/brainstem tissue using genome-wide unbiased transcriptome profiling. Promising candidates were validated with quantitative immunoblots.

## Results

### Overexpression of A53T-Alpha-Synuclein Modulates *Foxp1*, *Cplx1, Rabl2a, Rabgef1* and *Ywhae* mRNA Levels in Mouse Midbrain/Brainstem

**Fig. 1:**
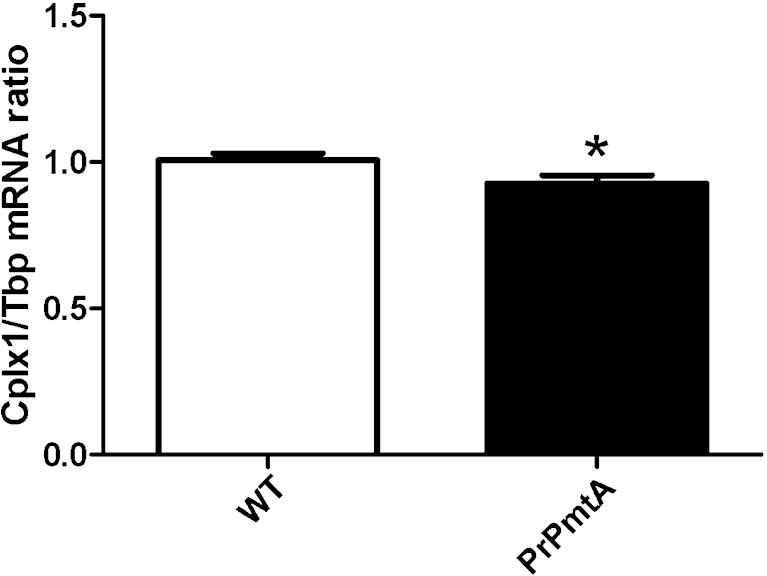
Quantitative real-time reverse transcriptase PCR demonstrates reduced mRNA levels of complexin-1 in the midbrain/brainstem of mice with A53T-alpha-synuclein overexpression. Tbp was always used as loading control, and mRNA level ratios were normalized to WT. *Asterisk* represents *p* value <0.05. Complexin-1 transcript was specifically detected by a custom-made Taqman assay, using midbrain/brainstem extracts from the transgenic line PrPmtA versus wild type (WT) (*n* = 18 versus 15) at age 18 months, demonstrating a significant downregulation in PrPmtA tissue

Previously documented (GEO database GSE20547, see also [12]) global transcriptome data from striatum, midbrain/brainstem and cerebellum of human A53T-alpha-synuclein overexpressing mice were filtered. We selected those significant changes at age 18 months relative to age 6 months, which were midbrain/brainstem-specific and were consistent between both transgenic mouse lines (PrPmtA and PrPmtB). Further selection prioritized those transcripts with no corresponding significant changes in wild-type midbrain/brainstem and in wild-type/transgenic striatum and cerebellum, resulting in the identification of 49 candidate effects of synucleinopathy (Table 1). Among the progressive upregulation effects, the increase of *Foxp1* mRNA levels by A53T-alpha-synuclein overexpression was particularly interesting in view of our previous finding that *Foxp1* (encoding forkhead box P1) is downregulated in alpha-synuclein knockout mouse [15]. Thus, the midbrain-identity-mediating transcription factor Foxp1 appears to depend in its brain levels both on the gain-of-function and the loss-of-function of alpha-synuclein. Among the progressive downregulation effects, the decreased levels of *Cplx1* (encoding complexin-1) selectively in the mutant midbrain/brainstem were especially interesting, in view of the co-localization of alpha-synuclein and complexin-1 at the SNARE complex. Other midbrain/brainstem-selective significant dysregulations of vesicle endocytosis/exocytosis pathway factors included the downregulation of *Rabl2a* transcript and the upregulation of *Rabgef1*, in good agreement with previous observations that alpha-synuclein disrupts cellular Rab homeostasis [16]. More ubiquitous dysregulations were detectable for three 14-3-3 isoforms (Table 1 bottom), which are established downstream targets of alpha-synuclein function. 14-3-3 family isoforms heterodimerize to protect phosphoserine-phosphothreonine groups and are (1) sequence homologous to alpha-synuclein, (2) putative direct interactor proteins of alpha-synuclein, (3) expression-modulated by alpha-synuclein, (4) sequestered into the Lewy bodies, (5) able to change their levels in response to pathogenic alpha-synuclein mutations and thereby modulate neurotoxicity [17–20]. Thus, at least seven of the expression changes are credible in the light of their function and of previous knowledge about alpha-synuclein. All information per animal including age and sex with the resulting microarray data discussed in this publication is deposited in the NCBI's database Gene Expression Omnibus (GEO) and is accessible through GEO series accession number GSE20547. Validation studies of *Cplx1* mRNA expression in independent tissues by qPCR confirmed the significant downregulation in midbrain/brainstem of PrPmtA mice (Fig. 1).

**Table 1:**
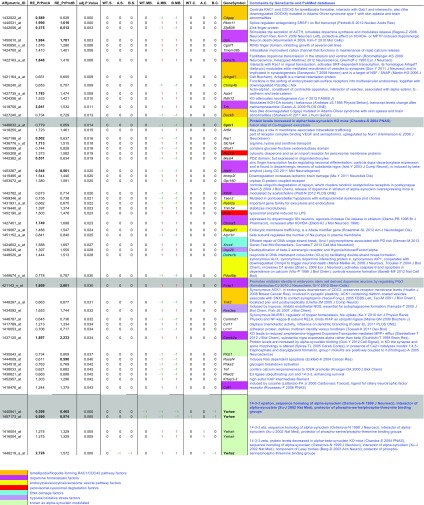
Global transcriptome analysis of mice with nigrostriatal overexpression of human A53T-alpha-synuclein showing significant changes from age 6 to 18+ months

The upper rows show all 49 genes with known functions, which exhibited significant and consistent progression changes in both transgenic midbrain/brainstem tissues, but not in wild-type midbrain/brainstem or striatum or cerebellum. Grey background with bold gene symbol and comments were used to highlight the most promising novel expression effect of synucleinopathy, *Cplx1* (encoding complexin-1). The lower rows show known expression effects of synucleinopathy for comparison, highlighting the best previously established transcript *Ywhae* (encoding 14-3-3epsilon). Column (**A**) documents the Affymetrix probeset ID; (**B**, **C**) the relative expression (RE) values for transgenic lines PrPmtA and PrPmtB, respectively, highlighting changes >1.7 or <0.6 in bold letters; (**D**) the adjusted *p* value to judge significance after correction for multiple testing; (**E–G**) the lack of significant changes (0) in striatum (S) of wild type (WT) and the two transgenic lines (A and B), respectively; (**H–J**) the lack of significant changes in WT compared to significant upregulations (1) or downregulations (−1) in midbrain/brainstem (MB) tissue of two transgenic lines A and B, respectively; (**K–M**) the lack of significant changes in cerebellum (C) of wild type and two transgenic lines A and B, respectively; (**N**) the gene symbol to access GeneCards and NCBI online databases using different background colours to emphasize functional pathways in common between individual genes; (**O**) authors’ summaries on the functions of each gene product with respect to synaptic failure, according to GeneCards and PubMed online databases. The rows of the upper table part were ordered from top in descending significance

### Overexpression of A53T-Alpha-Synuclein Leads to Elevated Complexin-1 Protein Levels in Mouse Midbrain/Brainstem

**Fig. 2:**
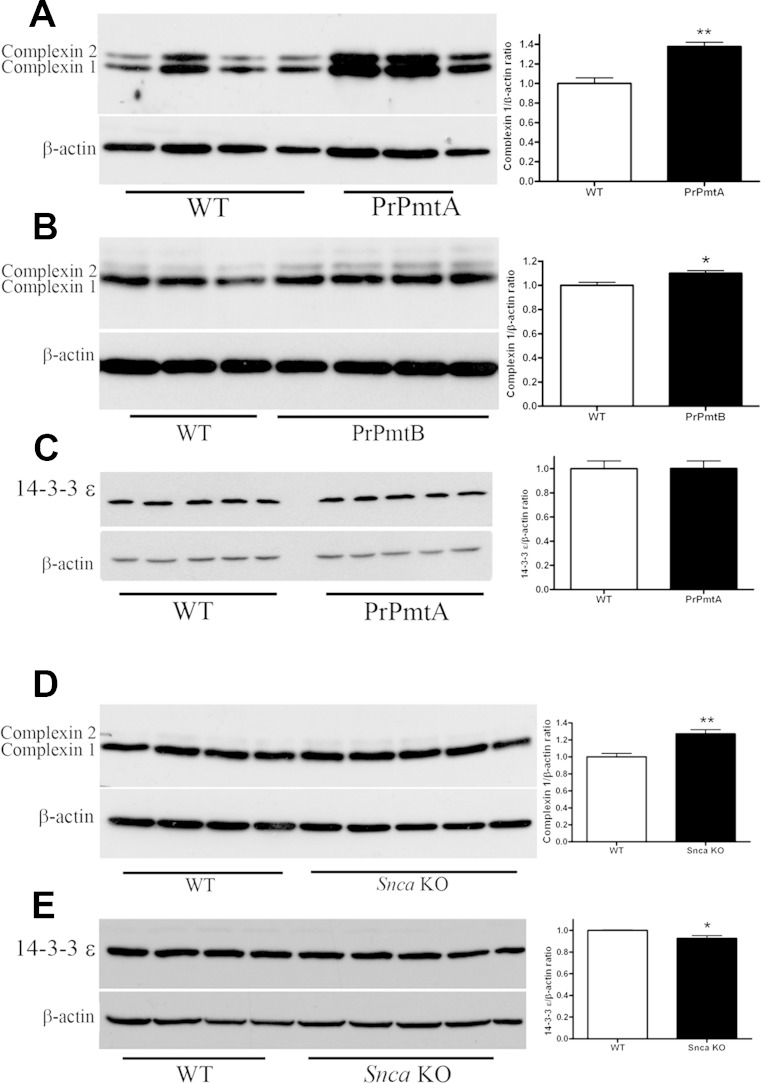
Quantitative immunoblots demonstrate dysregulated levels of complexin-1 and 14-3-3epsilon proteins in the midbrain/brainstem of mice with alpha-synuclein mutation. Beta-actin was always used as loading control, and protein level ratios were normalized to WT. Representative membranes are shown at the *left*, *bar graph statistics* of quantification at the *right*. *p value <0.05, **p < 0.01 and ***p < 0.001. **a** Complexin-1 and complexin-2 were detected with the antibody from SySy, using midbrain/brainstem protein extracts from the transgenic line PrPmtA versus wild type (WT) (*n* = 3 versus 4) at age 18 months, demonstrating significantly increased complexin-1 levels. **b** Midbrain/brainstem protein from transgenic line PrPmtB versus wild type (WT) (four vs. three) at age 18 months also showed significantly increased complexin-1 levels. **c** In comparison, selective detection of 14-3-3epsilon abundance change (five vs. five) as a repeatedly published molecular effect of alpha-synucleinopathy failed to reveal changes in protein levels, in spite of its significantly changed mRNA levels in mouse midbrain/brainstem (Table 1). **d** Levels of complexin-1 and complexin-2 (antibody from SySy) were significantly increased in alpha-synuclein knockout mice (*Snca* KO) at age 3 months (five KO vs. four WT), in inverse correlation to alpha-synuclein levels, demonstrating that complexin levels respond not only to the toxic alpha-synuclein gain-of-function/aggregation process but also to its loss-of-function. **e** Significant downregulation of 14-3-3epsilon (five KO vs. four WT). These data indicate that 14-3-3epsilon protein levels are directly correlated to the loss-of-function of alpha-synuclein

We focused on the downregulation of midbrain/brainstem *Cplx1* mRNA as a novel and promising effect, since the encoded protein complexin-1 is involved in the stimulus-dependent control of secretory vesicle exocytosis through the SNARE complex [21, 22]. Alpha-synuclein was also shown to modulate SNARE assembly and vesicle clustering, so this expression effect might constitute a very direct and early consequence of alpha-synuclein mutations. Densitometric analysis of immunoblots revealed a significant increase of complexin-1 protein levels in the midbrain/brainstem of aged A53T-alpha-synuclein overexpressing mice (Fig. 2a–c), despite *Cplx1* mRNA downregulation. The alterations were readily apparent by ECL detection of membranes, making more sophisticated approaches such as near-infrared immunoblot detection or quantification by ELISA unnecessary.

### Deficiency of Alpha-Synuclein Also Modulates the Complexin-1 Levels in Mouse Midbrain/Brainstem

These data obtained in A53T-alpha-synuclein overexpressing midbrain/brainstem complement previous observations from alpha-/beta-synuclein double-null mice, which exhibit upregulated complexin-1 and downregulated 14-3-3 epsilon protein in the whole brain [23]. To test whether alpha-synuclein or beta-synuclein is responsible for the observed changes, we studied midbrain/brainstem from mice with *Snca* knockout in 129/SvEv background [24] and demonstrated significant upregulation for complexin-1 and downregulation for 14-3-3epsilon protein (Fig. 2d–e). Thus, 14-3-3 isoform and complexin-1 protein levels respond not only to toxic gain-of-function mutations in alpha-synuclein but also to its loss-of-physiological function.

## Discussion

Our data confirm previous findings that alpha-synuclein abundance modulates the levels of 14-3-3 isoforms. It was previously known that CPLX1 levels are altered in alpha + beta-synuclein double-knockout mice and that *Foxp1* mRNA levels respond to the alpha-synuclein knockout. We now report novel findings that also pathogenic gain-of-function mutations of alpha-synuclein have a modulatory role on CPLX1 and *Foxp1* in mice that showed no demonstrable alpha-synuclein aggregation in midbrain/brainstem during their lifespan and that CPLX1 levels change in the alpha-synuclein single knockout mouse brain.

This suggests that both CPLX1 and FOXP1 may be useful to monitor early stages of alpha-synuclein pathology. FOXP1 is expressed preferentially in the midbrain. In contrast, CPLX1 shows a more ubiquitous expression pattern, similar to alpha-synuclein. Although both CPLX1 and SNCA were mainly studied regarding presynaptic vesicle dynamics, their expression in brain is not much higher than in blood platelets, where they have a role in stimulus-dependent secretory vesicle exocytosis to control thrombus formation [25]. Thus, CPLX1 might have potential as biomarker to monitor an alpha-synuclein gain-of-function in peripheral tissues like blood.

Although our experiments were focused on modelling monogenic alpha-synucleinopathy variants of PD (PARK4/1), we are confident that complexin-1 plays a role in the genetically heterogeneous common idiopathic PD. Our data from alpha-synucleinopathy mouse models are consistent with a proteome survey of midbrain from sporadic PD patients, which reported significantly elevated levels for complexin-1 and a trend towards elevated levels of 14-3-3 epsilon [26]. Furthermore, the *GAK*/*CPLX1* locus on chromosome 4 carries risk variants for sporadic PD in GWAS studies [6].

The accumulation of CPLX1 in spite of reduced *Cplx1* mRNA levels is intriguing. A plausible explanation might predict that excess alpha-synuclein at the SNARE complex interacts with CPLX1 and impairs its degradation. This could occur as part of a sequestration process during the formation of alpha-synuclein oligomers and aggregates, reflecting incipient formation of inclusion bodies known as “Lewy neurites” or “Lewy bodies”. It has been observed that this aggregation process starts in the presynapses and sequesters local proteins such as synapsin [27]. Overall, the transcriptomic profiling of our PARK1/PARK4 mouse model identified plausible molecular correlates of early nigrostriatal dopaminergic neurotransmission deficits previously observed in this mouse [9].

In conclusion, the transcriptomic profiling of mouse midbrain/brainstem tissue with alpha-synuclein pathology has provided credible insights into early steps of pathogenesis, before the advent of neurodegeneration. Complexin-1 may be a better read-out of alpha-synucleinopathy than the previous gold standard 14-3-3.

## Materials and Methods

### Ethics Statement

Mice were housed in accordance with the German Animal Welfare Act, the Council Directive of 24 November 1986 (86/609/EWG) with Annex II, the ETS123 (European Convention for the Protection of Vertebrate Animals) and the EU Directive 2010/63/EU for animal experiments at the FELASA-certified Central Animal Facility (ZFE) of the Frankfurt University Medical School.

*Mouse breeding and characterization* with brain dissection was carried out as described in the literature [28, 29, 24]. All studies of mouse mutants were in comparison with age- and sex-matched WT controls from the same inbred background line, which were bred and aged in parallel, under controlled light cycle, periodic health-monitoring, and individually ventilated cage housing. Dissection of brain regions occurred rapidly after cervical dislocation, placing the brain in a sagittal view to perform a coronal section from the hypophysis stem towards the caudal end of the hippocampus. Olfactory brain regions, the cerebral cortex, septal and thalamic tissue were removed from the ventral tissue block to isolate the striatum. To obtain midbrain/brainstem from the caudal tissue block, the cortical, dorsal thalamic and tectal tissues were removed, yielding the substantia nigra continuous with ventral tegmental area, red nucleus, mammillary nuclei and brainstem. For the dissection of the cerebellum, its peduncles were cut at the entry points into the hindbrain. All tissues were snap-frozen in liquid nitrogen and then stored at −80 °C. Extraction of protein and RNA was carried out as previously described [30]. The individual transcript expression validation on a StepOnePlus equipment (AppliedBiosystems) employed TaqMan assays (AppliedBiosystems) Mm00447333_m1 (*Snca*), Mm01198853_m1 (*Cplx1*) and Mm00446973_m1 (*Tbp*), with quantitative real-time reverse transcriptase polymerase chain reaction (qPCR) conditions as recommended for these assays.

*Genome-wide transcriptomics* of mouse brain regions was performed with Affymetrix oligonucleotide microarrays as previously reported [12].

### Quantitative Immunoblots

Frozen tissues were homogenized on ice in a glass-Teflon douncer in RIPA buffer with 50 mm Tris–HCl (pH 8), 150 mm NaCl, 1 % NP-40, 0.5 % Na-deoxycholate, 0.1 % SDS and protease inhibitor cocktail (Roche). Total lysates were briefly sonicated on ice, and cell debris was removed by centrifugation. Protein concentration was determined according to the method of Bradford. SDS–PAGE-separated proteins (20 μg/lane) were blotted onto a PVDF membrane (Bio-Rad) and probed. The following primary antibodies for mouse alpha-synuclein (1:1,000 BD Biosciences 610786), complexin-1 (1:500 Acris AP51050PU-N and 1:1,000 SySy 122002), 14-3-3epsilon/eta/zeta/beta/gamma/theta (1:1,000 SantaCruz sc1020 and others from CellSignaling), beta-actin (1:1,000 Sigma A5441) were used with their corresponding secondary antibodies (GE Healthcare UK Limited LNA931V/AG for ECL-anti-mouse-HP from sheep and LNA934V/AG for ECL-anti-rabbit-HP from donkey) for 1 h. The detection was made with SuperSignal West Pico (Thermo Scientific), with varying exposure times to avoid film sensitivity or saturation problems as well as non-linear effects. The images were digitalized on a scanner (Epson) and densitometry performed with the proprietary ImageMaster Total Lab 2.00 software (AmershamPharmacia) or the public ImageJ software. After normalization of candidate protein values versus beta-actin values from the identical membrane in EXCEL, the changes were evaluated in GraphPad statistics and plotting.

*Statistical analyses* presented in bar graphs were performed by unpaired Student’s *t* tests and plotted with the Prism 3 software (GraphPad, La Jolla, CA, USA).
